# Peripheral Lymphadenopathy: Approach and Diagnostic Tools

**Published:** 2014-03

**Authors:** Shahrzad Mohseni, Abolfazl Shojaiefard, Zhamak Khorgami, Shahriar Alinejad, Ali Ghorbani, Ali Ghafouri

**Affiliations:** 1 Department of Surgery, Shariati Hospital, Tehran University of Medical Sciences, Tehran, Iran;; 2 Research Center for Improvement of Surgical Outcomes and Procedures, Department of Surgery, Shariati Hospital, Tehran University of Medical Sciences, Tehran, Iran

**Keywords:** Lymphadenopathy, Localization, Benign, Malignant, Diagnosis

## Abstract

Peripheral lymph nodes, located deep in the subcutaneous tissue, clean antigens from the extracellular fluid. Generally, a normal sized lymph node is less than one cm in diameter. Peripheral lymphadenopathy (LAP) is frequently due to a local or systemic, benign, self-limited, infectious disease. However, it could be a manifestation of underlying malignancy. Seventy-five percent of all LAPs are localized, with more than 50% being seen in the head and neck area. LAP may be localized or generalized. Cervical lymph nodes are involved more often than the other lymphatic regions. Generally, it is due to infections, but most of the supraclavicular lymphadenopathies are associated with malignancy. Based on different geographical areas, the etiology is various. For example, in tropical areas, tuberculosis (TB) is a main benign cause of LAP in adults and children. Complete history taking and physical examination are mandatory for diagnosis; however, laboratory tests, imaging diagnostic methods, and tissue samplings are the next steps. Tissue diagnosis by fine needle aspiration biopsy or excisional biopsy is the gold standard evaluation for LAP.

We concluded that in patients with peripheral LAP, the patient’s age and environmental exposures along with a careful history taking and physical examination can help the physician to request step by step further work-up when required, including laboratory tests, imaging modalities, and tissue diagnosis, to reach an appropriate diagnosis.

## Introduction

The human body has about 600 lymph nodes.^1^ Spleen, tonsils, adenoids, and Peyer's patches are parts of the lymphoid tissue, and their role is to clean antigens from the extracellular fluid. Peripheral lymph nodes are those which are located deep in the subcutaneous tissue and can be palpated if any process causes them to enlarge. Lymphadenopathy (LAP) is the term to describe the conditions in which lymph nodes become abnormal in size, consistency, and number.

A normal sized lymph node is usually less than one cm in diameter. Of course, there are exceptions in lymph nodes in different regions and at different ages have different sizes. For example, some authors have proposed that an inguinal lymph node size up to 1.5 cm should be considered normal, while the normal range for the epitrochlear nodes is up to 0.5cm.^2^ In general, normal lymph nodes are larger in children (ages 2-10), in whom a size of more than 2 cm is suggestive of a malignancy (i.e., lymphoma) or a granulomatous disease (such as tuberculosis or cat scratch disease).^3^

It is important to take a careful history to consider a variety of disorders, which may be a clue to the underlying disorder. It might be a usual self-limited infection in younger adults or a malignancy in older patients. Based on different geographical areas, the etiology varies. For example, tuberculosis (TB) is the most common cause of cervical LAP in endemic areas such as Africa.^4^^-^^8^ Nonetheless, in a large number of studies, the most common benign etiologies are non-specific reactive changes in lymph nodes.^9^^-^^11^

Despite the low prevalence of malignancy among patients with LAP, it remains to be the main concern of both patients and physicians. Studies have shown that its prevalence is less than one percent among patients with unexplained LAP in general practice.^12^

Several aspects in the diagnosis of LAP should be considered. In most cases, further investigation is not required as the cause is obvious on primary evaluation (such as infection). In unexplained conditions, laboratory tests, imaging studies, and tissue biopsy are recommended. Imaging can identify the size and distribution of the node more accurately than can physical examination. Ultrasound is a noninvasive method to assess lymph nodes in superficial regions like the neck.^13^ Computed tomography (CT) is useful to determine LAP in the thorax or abdominopelvic cavity.^14^^,^^15^ Tissue diagnosis by fine needle aspiration biopsy or excisional biopsy is the gold standard evaluation for LAP.^16^

Several articles have discussed the appropriate approach to the diagnosis and management of LAP. In this article, we discuss various aspects of peripheral LAP and describe how a physician can approach it. In order to provide a comprehensive review of various aspects of peripheral LAP, we performed comprehensive literature search and review through electronic databases, including PubMed, Elsevier, Scholar Google, IranMedex, and Scientific Information Database (SID), using “peripheral lymphadenopathy”, “localization”, “benign”, “malignant”, and “diagnosis” for articles published between 1984 and 2011.

## Epidemiology

In tropical areas, TB is a main benign cause of LAP in adults and children.^4^^,^^5^^,^^17^^,^^18^ In patients with TB, the assessment of the human immunodeficiency virus (HIV) is advised because it increases the incidence of extrapulmonary TB to more than 50%.^19^^-^^21^Infectious mononucleosis affects patients of all ages; however, it is more frequent before adolescence. Approximately over 90% of adults all over the world are seropositive for this viral disease, although only 25-30% of them have become clinically ill.^14^^,^^22^

In general practice, less than one percent of patients with LAP have malignant disease,^12^ often due to leukemia in younger children and Hodgkin's disease in adolescents.^23^ It has been reported that the prevalence of malignancy is 0.4% in patients under 40 years and 4% in those over 40 years of age in the primary care setting.^14^ The prevalence rises to 17% in referral centers^15^and soars to 40-60% in highly suspicious patients.^14^ Be that as it may, the location of LAP changes the possibility of malignancy.

Hodgkin's disease is rare before 10 years old and a small male dominance is present, especially in childhood. The Epstein-Barr virus infection in combination with immune deficiency is a risk factor for increasing Hodgkin's disease, particularly in less-developed countries and low socioeconomic conditions. Non-Hodgkin's lymphoma, the fourth common worldwide malignancy in males with a frequency of 6.1%,^24^ is another cause.

## History Taking

Taking a complete history of the patient is necessary to determine the etiology of LAP. Age, time of presentation, duration of symptoms, underlying diseases, and circumstances in which LAP was detected are of great value. Furthermore, a history of exposure to animals, ingestion of certain drugs and foods, risky behaviors, and history of recurrent infection and immunodeficiency can help the diagnosis. 

A history of environmental exposure to tobacco, alcohol, and ultraviolet radiation increases the suspicion of the metastatic carcinoma of the internal organs, head, and neck as well as skin malignancies. Immune deficient patients, like those with AIDS, have wide differential causes of LAP and malignancies like Kaposi’s sarcoma; however, non-Hodgkin's lymphoma should always be taken into consideration.^16^

A family history of malignant disorders may raise the physician’s suspicion to distinct etiologies of LAP such as breast carcinomas, melanoma, and dysplastic nevus syndrome.^16^

Also, if LAP lasts less than two weeks or over one year without increasing in size, the probability of malignancy is quite low.^16^

## Related Symptoms and Signs

A recent upper respiratory infection can cause cervical LAP, which is usually self-limited. A triad of moderate to high fever, pharyngitis, and moderately tender lymph node with splenomegaly (>50%) characterizes classic infectious mononucleosis.^25^Cytomegalovirus, toxoplasmosis, HIV, and human herpes virus type 1 can cause mononucleosis-like syndrome.^25^The typical symptoms of toxoplasmosis are flu-like symptoms, with a single swollen cervical lymph node.^14^^,^^16^HIV in the acute phase presents with mononucleosis-like syndrome. Its presentation consists of fever, fatigue, pharyngitis, rash, malaise, arthralgia, and LAP, which appear 2-6 weeks after exposure to the HIV virus.^26^^,^^27^

A recent travel to an endemic area or exposure to an infected patient with TB along with painless, gradually progressive, single or matted lymph nodes can suggest mycobacterium TB involvement.^28^The coexistence of LAP and symptoms like arthralgia, muscle weakness, unusual rash, and anemia may direct the diagnosis of autoimmune diseases, including rheumatoid arthritis, systemic lupus erythematous, and dermatomyositis.^1^^,^^16^ On the other hand, whenever dermatomyositis is diagnosed, the underlying malignancy should be ruled out.

Significant fever, night sweats, and unexplained weight loss (more than 10% in less than 6 months) are the “B symptoms” of lymphoproliferative disorders, but they may also be seen in TB or collagen vascular diseases.^29^

Petechiae and purpura associated with LAP and splenomegaly may be detected in acute leukemias.^30^Pain may occur in involved nods with Hodgkin's disease following alcohol consumption.^29^ Generalized pruritus is a concerning symptom because it manifests in 30% of patients with Hodgkin's disease^31^ and 10% of patients with non-Hodgkin's lymphoma.^32^

## Physical Examination

All patients with LAP should undergo a complete and systematic physical examination. Any palpable lymph node should be evaluated for its location, size, consistency, fixation, and tenderness. 

*Location*

Determining whether LAP is localized or generalized makes the differential range narrower. An enlarged node in a lymphatic-rich region mostly presents a local disease. The presence of a red lymphangitic streaking (lymphangitis) may be detected in a localized infection.^33^

Nodes that are associated with malignancy tend to involve several groups of nodes. ^34^

LAP in the supraclavicular area has the highest risk of malignancy; this risk is 90% in patients more than 40 years old and 25% in those under 40 years old.^12^ The Virchow node, in the left supraclavicular area, suggests intra-abdominal malignancies (e.g., gastric carcinoma), while in the right side suggests intra-thoracic malignancies.

*Size*

It is suggested that palpable supraclavicular, iliac and popliteal nodes, epitrochlear greater than 0.5cm, and inguinal nodes larger than 1.5 cm are abnormal.^16^ The nodes in other areas are considered as abnormal if their diameter exceeds one cm.^2^ However, there is no uniform nodal size at which the greater diameter can raise suspicion of a neoplastic etiology.

*Pain and Tenderness*

Pain and tenderness on a lymph node is a non-specific finding. It is typically due to infection. In some cases, pain is induced by hemorrhage into the necrotic center of a neoplastic node, immunologic stimulation of pain receptors, or rapid tumor expansion.^12^

*Consistency*

Acute inflammation by infiltrating the node may make it more consistent, with concomitant tenderness due to the tension on the capsule. Chronic inflammation also leads to fibrotic changes, making the node hard in palpation. Stony-hard and painless nodes are usually signs of metastatic cancer or granulomatous disease. Firm and rubbery nodes can imply lymphoma. Matted lymph nodes are described when a group of nodes are conglomerated. They can be either due to benign (mycobacterial infection and sarcoidosis) or malignant (lymphoma and metastatic carcinoma) disorders.^1^^,^^16^^,^^35^

*Mobility*

LAPs resulting from infections and collagen vascular diseases are usually freely movable in the subcutaneous region. Rubbery mobile nodes are associated with lymphoma. Nodes that are associated with malignancy are often fixed to the skin or surrounding tissues.^36^^,^^37^

Organomegaly (especially splenomegaly) is sometimes associated with LAP, as in infectious mononucleosis, acute lymphoma, Hodgkin's disease, non-Hodgkin's lymphoma, and sarcoidosis.^29^

Skin should also be examined for unusual lesions suggesting malignancy such as melanoma, and for traumatic lesions that potentially can be an inoculation site for microbial germs.

## Classification and Etiology

Seventy-five percent of all LAPs are localized, and more than 50% are detected in the head and neck area. They are often caused by a specific pathology in the region of the lymphatic drainage, which can be diagnosed without additional assessment. Twenty-five percent of LAPs are generalized and are often a sign of a significant systemic underlying disease.^14^ There are a variety of etiologies which can lead either to localized or generalized LAP (table 1).^16^^,^^29^^,^^36^

**Table 1:** Differential Diagnosis of Peripheral Lymphadenopathy

**Localized Peripheral Lymphadenopathy**	
Cervical	*Infections:*
	Viral: Upper respiratory tract infections, mononucleosis, herpes virus, coxsackie virus, cytomegalovirus, HIV
	Bacterial: Staphylococcus aureus, Streptococcus pyogenes (group A), mycobacterium, dental abscess, cat scratch disease
	*Malignancy:* Hodgkin's disease, non-Hodgkin's lymphoma , thyroid cancer, squamous cell carcinoma of the head and neck
Supraclavicular	*Malignancy*: Abdominal/thoracic neoplasm, thyroid cancer, Hodgkin's disease, non-Hodgkin's lymphoma, breast carcinoma
	*Infections: *Mycobacterial, fungal
Axillary	*Infections*: Staphylococcal and Streptococcal skin infections, cat scratch disease, sarcoidosis
	*Malignancy: *Breast cancer, lymphomas, luekemias
Inguinal	*Benign Reactive Lymphadenopathy*
	*Infections*: Sexually transmitted disease, cellulitis
	*Malignancy*: Lymphomas, squamous cell carcinoma of the penis and vulva, metastatic melanoma
Generalized Peripheral Lymphadenopathy	
Infections	Mononucleosis, HIV, miliary tuberculosis, typhoid fever, syphilis, plague
Malignancy	Lymphomas, acute leukemias
Autoimmune Disorders	Systemic lupus erythematosus, rheumatoid arthritis, Sjögren’s syndrome, sarcoidosis
Drug Reactions	Phenytoin, Allopurinol, Atenolol
Lipid Storage Diseases	Gusher's disease, Neiman-Peak

The data of the table are derived from references cited in the text.

## Localized Adenopathy

Cervical lymph nodes are involved more often than are other lymphatic regions. They also have an extensive range of differential diagnoses, making the approach more important. Bacterial or viral infection of the face, nasopharynx, or oropharynx is the most common cause of cervical LAP.^38^Generalized LAP caused by viruses like Ebstein-Barr Virus and cytomegalovirus, may also present with acute bilateral cervical lymphadenitis.^39^ Acute pyogenic lymphadenitis, usually due to skin infection by *Staphylococcus aureus* or pharyngitis by group A *Streptococci*, is more common in children. TB also involves the cervical lymph node in 60% to 90% of cases;^21^ they are firm and non-tender and are known as atypical TB.^21^ Cat scratch disease, also known as sub-acute regional lymphadenitis, is caused by *Bartonella henselae*, a Gram-negative bacterium. LAP is seen in more than 80% of these patients.^40^ Hodgkin's disease, non-Hodgkin's lymphoma, and squamous cell carcinoma of the head and neck and metastatic carcinomas are common malignancies in the cervical region.^16^^,^^41^ Papillary and follicular thyroid cancer and nasopharyngeal carcinomas can also involve and metastasize to the cervical lymph nodes.^38^ Clinical cervical LAP has been found in 15-30% of the cases of papillary thyroid carcinoma.^42^

Supraclavicular LAPs, associated with malignancy in all ages, should always be investigated even in children. The right supraclavicular lymph nodes drain the mediastinum, lungs, and esophagus, while the left nodes drain the gastrointestinal tract and genitourinary tract, which can be involved with the malignancy of these organs. Hodgkin's disease, non-Hodgkin's lymphoma, breast carcinoma, mycobacterial, and fungal infections can also involve the lymph nodes of this region.^29^

Axillary LAP is most commonly non-specific or reactive.^16^ The anterior and central axillary lymph nodes may be palpable due to breast cancer metastasis even before the main lesion is detected. Hodgkin's disease and non-Hodgkin's lymphoma are seldom seen solely in the auxiliary nodes.^16^ Cat scratch disease also is a common cause of axillary LAP.^40^

Benign reactive inguinal LAP is seen in patients who walk barefooted outdoors. Localized LAP is typically caused by infection and is due to sexual transmitted diseases (herpes simplex virus, gonococcal infection, syphilis, chancroid, granuloma inguinale, and lymphogranuloma venereum). Malignancy rarely presents itself only in the inguinal lymph nodes. Occasionally, Hodgkin's disease, non-Hodgkin's lymphoma, melanoma, and squamous cell carcinoma of the penis, vulva, and anus can involve the lymph nodes of this region.^16^

## Generalized Adenopathy

The etiology of generalized adenopathy may sometimes overlap with localized LAP (table 1)^16^^,^^29^^,^^36^and almost always indicates an underlying disease. Some important and common causes are as follows:

The Epstein-Barr virus typically involves the bilateral posterior cervical, axillary, and inguinal lymph nodes, distinguishing it from the other causes of pharyngitis. LAP appears in the first week of exposure and then gradually subsides over two to three weeks. Low-grade fever, fatigue, and prolonged malaise are the other symptoms.^25^

HIV infection is frequently associated with generalized LAP. It may also increase the risk of TB. The HIV initially involves the cervical, auxiliary, and occipital nodes and is not tender.^43^ In this situation, lymph nodes enlargement lasts more than 2-3 months.^14^ Drug reaction is characterized by fever, rash, arthralgia, and generalized LAP.^16^^,^^29^

Generalized lymph node enlargement is a common and is usually a non-specific aspect of systemic lupus erythematosus. It is frequently detected in the cervical, axillary, and inguinal regions. Whereas lymph node necrosis is the characteristic histological finding, reactive follicular hyperplasia is the most frequent histopathologic finding in lymph node lesions in systemic lupus erythematosus patients.^44^

Generalized LAP is rarely seen in malignancies; however, it is usually seen in non-Hodgkin's lymphoma, whereas Hodgkin's disease is distinguished by the localized involvement of the lymph nodes.^30^

## Differential Diagnosis

Three models are available to categorize peripheral LAP.

1. Using the acronym "CHICAGO" helps to consider all causes.^29^
**C→**Cancers**: ***Hematologic malignancies*: Hodgkin's disease, Non-Hodgkin's lymphoma, Leukemia
*Metastatic*: Breast tumor, Lung, Kidney, others
H**→**Hypersensitivity syndromes: Serum sickness, Drugs
I**→**Infections: Viral (Epstein-Barr virus, cytomegalovirus, HIV), Bacterial (TB,) Fungal, Protozoan, Rickettsial (Typhus), Helminthes
C**→**Connective Tissue disorders: Systemic lupus erythematosus, Rheumatoid arthritis, Dermatomyositis
A**→**Atypical lymphoproliferative disorders:  Castleman’s Disease, Wegener
G**→**Granulomatous: Histoplasmosis, Mycobacterial infections, Cryptococcus, Berylliosis, Cat scratch disease, Silicosis
O**→**Others
2. Using the letters of alphabet, although it makes the categorization too long.^29^
3. Using the region of lymph node enlargement and its localization provides useful information about causes.^29^

## Diagnostic Approach

**Figure 1:**
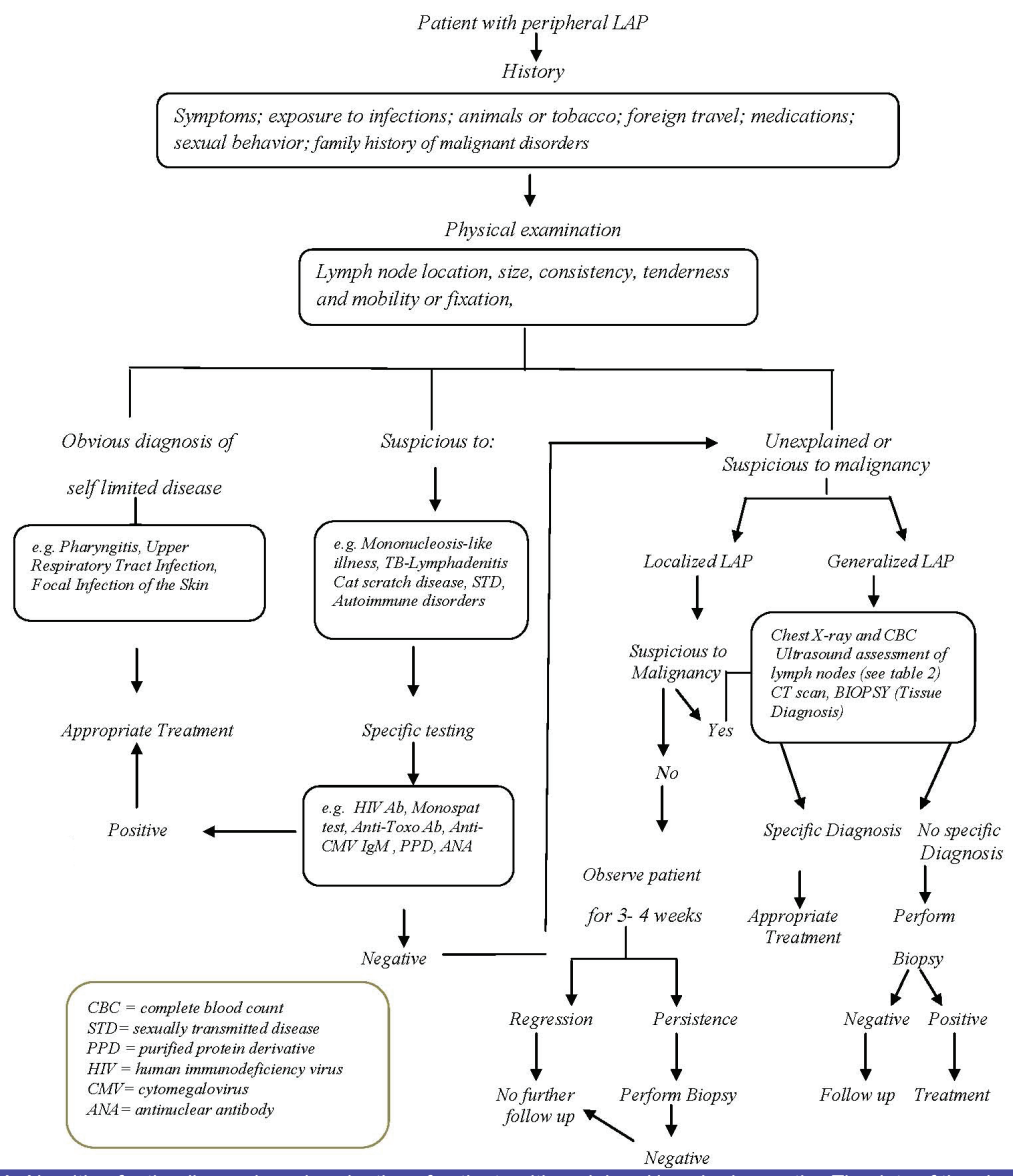
Algorithm for the diagnosis and evaluation of patients with peripheral lymphadenopathy. The data of the algorithm are derived from references cited in the text.

Following comprehensive history taking and physical examination, the existing algorithm (figure 1) can guide the physicians for a further evaluation of patients with peripheral LAP.^1^^,^^14^^,^^16^

*Laboratory Diagnostic Methods*

If more work-up is needed, the first step is to obtain complete blood count (CBC).

In bacterial pharyngitis, a throat culture or rapid antigen detection tests is helpful. Lymphocytosis (>50% of leukocytes) with the presence of at least 10% atypical lymphocytes and a positive serologic test of the Epstein-Barr virus are typical laboratory findings in the Epstein-Barr virus involvement.^14^ However, the presence of atypical lymphocytes in a peripheral blood smear can be due to acute leukemia,^35^ which mandates further evaluations such as bone marrow biopsy.^16^Anti-cytomegalovirus IgM antibodies or cytomegalovirus polymerase chain reaction (PCR) are laboratory tests for diagnosing cytomegalovirus.^14^Anti-HIV antibodies reach detectable levels about two weeks after infection, and HIV PCR can be helpful in this phase.^14^ IgM toxoplasma antibody is the diagnostic serologic test for the acute phase infection of toxoplasmosis.^1^

If autoimmune diseases are suspected, CBC, antinuclear antibody, dsDNA antibody, ESR, and rheumatoid factor and complement level should be checked. Lymphocytosis can be seen in leukemia, autoimmune disorders, Epstein-Barr virus, cytomegalovirus, and TB. Increased neutrophil count in CBC is detected in acute bacterial infection. An extreme increase in the total number of leukocytes (more than 50000 WBC/mm^ 3 ^) is a leukemoid reaction. It can be found in response to an infection (such as acquired immune deficiency syndrome), inflammation, and rarely in myeloproliferative disorders (i.e., chronic myelocytic leukemia).^45^

The existence of anemia (or other cytopenias) implies a significant underlying illness.^35^ Leukemia, HIV, and systemic lupus erythematous may be accompanied by pancytopenia. Full blood count with hemogram, ESR, CRP, and LDH are helpful in diagnosing malignancies and autoimmune processes.

Since thorough history taking and physical examination can lead to request for further work-up, figure 1 shows a step-by-step evaluation of and approach to patients with peripheral LAP.^1^^,^^14^^,^^16^

## Imaging

Imaging can identify node characteristics more accurately than can physical examination. Ultrasonography is a useful imaging tool in the assessment of the number, size, site, shape, margins, and  internal structure in patients with peripheral LAP, whereas CT scan and magnetic resonance imaging (MRI) are more useful in the evaluation of the thoracic and abdominopelvic cavity and their accuracy mainly depends on the size of the lymph nodes.^13^^,^^46^ Color Doppler ultrasonography has been used in the assessment of lymph node enlargement since the beginning of the 1970s.^47^^,^^48^ It can evaluate the vascular pattern, displacement of vascularity, vascular resistance, and pulsatility index. Hence, it can distinguish between an old LAP and a recent LAP that is still active.^49^^-^^51^ A normal or reactive node is usually oval with a hilum, whereas metastatic and lymphomatous lymph nodes generally emerge as round lesions.^46^^,^^52^Several studies have indicated that a low long axis to short axis of lymph nodes (L/S ratio) is a significant sign of lymphoma and metastatic cancer.^50^^,^^53^^-^^55^

Steinkamp HJ et al.^56^ detected the L/S ratio less than 2 was indicative of metastatic lymph nodes with 95% accuracy. But there is not any cut-off value for distinguishing the exact cause. Therefore, the L/S ratio is one of the parameters in the evaluation of lymph nodes and as such should be considered with other findings to reach a diagnosis.^51^ Metastatic nodes are often hypoechoic^50^^,^^51^^,^^57^^,^^58^ in comparison to the adjacent tissues. The absence of hilum has been reported in 76-96% of malignant nodes.^46^^,^^59^^,^^60^The ultrasonographic characteristics of benign and neoplastic LAPs are summarized in table 2.^46^^,^^56^^,^^61^

**Table 2:** Ultrasonographic Criteria of Benign and Neoplastic Lymphadenopathy

	**Shape**	**Border**	L/s Ratio*	**Internal Echogenicity**	**Hilum**	RI^**^	PI^***^	**Blood Flow Distribution**
Benign Disorders	ovoid	various	High>2	Isoechoic	Present-Normal	Low<0.8	<1.5	Hilar
Neoplastic Disorders	Round	sharp^†^	Low<2	Hypoechoic	Absent	High> 0.8	>1.5	Peripheral or miscellaneous

*Long axis to short axis (L/S); **Resistive index (RI); ***Pulsatility index (PI); ^†^In matted lymph nodes, the border is not sharp. The data of the table are derived from references cited in the text.

The resistive index and the pulsatility index, vascular resistance indices measured by spectral Doppler ultrasound, are useful to distinguish malignant from benign node disorders. Some studies have reported that malignancies in nodes tend to have a higher resistive index (>0.8) and pulsatility index (>1.5) than do reactive nodes.^47^^,^^61^^,^^62^However, other reports have posited that metastatic nodes have lower or similar vascular resistance compared with benign nodes.^50^^,^^63^ According to these various reports, the role of vascular resistance in the assessment of LAP is still controversial.

Some studies have suggested using patterns of vascular distribution within the nodes to distinguish benign from malignant nodes.^64^^-^^66^ Normal nodes usually have hilar vascularity. Reactive nodes tend to have more prominent hilar vascularity due to an increase in the blood flow.^61^^,^^67^Metastatic lymph nodes often have a peripheral perfusion pattern and abnormal hilar structure.^53^^,^^66^^,^^68^

In ultrasound assessment, microcalcification may be detected in 50-69% of the cases of papillary thyroid carcinomas.^53^ Microcalcification in metastatic axillary nodes is rare, but it strongly suggests breast cancer.^46^ Multiple lymph nodes, fusion tendency, and strong internal echoes (due to calcification) are the ultrasound characteristics of tubercular lymphadenitis.^51^^,^^69^

## Tissue Diagnosis

Tissue diagnosis is the gold standard in the evaluation of LAP. Fine needle aspiration cytology (FNAC) is a simple and safe procedure and is proved to be accurate in the diagnosis of reactive hyperplasia, infections, granulomatous lymphadenopathies, lymphomas, and metastatic malignancies. It is most helpful when looking for the recurrence of a previously diagnosed cancer. It is easily performed in both inpatient and outpatient settings and yields results promptly.^70^ The accuracy of diagnosing metastatic carcinoma in lymph nodes by FNAC is 82-96%.^71^^-^^73^ Using ancillary techniques like immunohistochemistry and flow cytometry improves the accuracy of FNAC for the diagnosis of lymphomas.^74^ FNAC has the maximum sensitivity and specificity for detecting metastatic cancers. Prasad et al.^72^ reported sensitivity of 97% and specificity of 98.9% in diagnosing metastatic lymph node by FNAC. The most important limitations of FNAC are inadequate specimen^75^ and high rate of false-negative diagnoses in Hodgkin's disease  and incomplete classification of non-Hodgkin's lymphoma.^70^

In patients suspected of LAP resulting from skin neoplasms (such as squamous cell carcinoma or melanoma), biopsy of the skin lesion is helpful.^16^

Ultrasonography-guided FNAC gives more precise information than does blinded FNAC because it guides the needle to the most suspicious area of the lymph node. Whenever physical examination and imaging techniques suggest malignancy, ultrasonography-guided FNAC can identify metastasis in the lymph node.^76^

Core needle biopsy, as another tissue diagnosis method, provides more specimen from the tissue than does FNAC. If an imaging technique guides the procedure, the results will be more accurate, and it may prevent unnecessary excisional biopsy.^77^ The accuracy of image-guided core needle biopsy in diagnosing lymphoma has been reported in the range of 76-100%.^41^^,^^78^^-^^84^

Percutaneous image-guided core needle biopsy is a safe and useful method for the diagnosis and classification of malignant lymphomas presenting with enlarged peripheral lymph nodes and superficial masses. It can be used as the first step for tissue sampling in a patient suspicious of lymphomas.^41^^,^^80^ Nevertheless, its strength for the diagnosis of lymphoma is still controversial and excisional biopsy of enlarged lymph nodes is regularly recommended as the gold standard procedure.^85^^,^^86^

Several approaches have been developed to recognize which patient with peripheral LAP needs excision biopsy. Vassilakopoulos et al.^87^ evaluated 475 patients older than 14 years old with LAP. They found that 6 variables among 23 examined clinical covariates independently predicted the need for lymph node biopsy, including age above 40 years, lack of tenderness on the lymph node, lymph node size, generalized pruritus, supraclavicular location, and hard texture of the lymph node. Ninety-six percent of the patients who needed biopsy were properly categorized by this model.

Oliver S. Soldes et al.^34^ suggested that some parameters increased the risk of malignancy in children more than 8 years old; these parameters were node size greater than one cm,  multiple sites of adenopathy, supraclavicular lymph nodes, fixed nodes, and abnormal chest X-ray. Moreover, the authors recommended that younger children with a single small node be preferably managed by laboratory tests and clinical follow-up because of the low risk of malignancy (≤5%).

Australian Cancer Network Diagnosis and Management of Lymphoma Guidelines, approved by the National Health and Medical Research Council (NHMRC), identified the following factors useful in determining the need for a lymph node biopsy:^88^ age more than 40 years; supraclavicular lymph node location; nodal diameter greater than 2.25 cm; firm-hard texture; and lack of pain.

## Histopathology

Based on the etiology, the histopathology of lymph nodes differs. We present a review of the salient points of some common diseases with regard to their histopathology.

Reactive LAP, which is the most common cause of lymph node enlargement, is a non-neoplastic and reversible enlargement of the lymphoid tissue secondary to antigen stimulus. There are five distinct patterns of benign LAP:^89^

1. * Follicular hyperplasia* is seen in infections, autoimmune disorders, and non-specific reactions. The histopathologic pattern is an increase in the size and number of the B-cells in the germinal center.
2. * Paracortical hyperplasia* is detected in viral infections, skin diseases, drug reactions, and non-specific reactions. The extension of the T-cells in the paracortical region is the pathologic pattern.
3. * Sinus hyperplasia* is seen in lymph nodes draining limbs due to inflammatory lesions and malignancies. The histopathologic pattern includes the expansion of the histiocyte cells in the medullary and cortical sinuses.
4. *Granulomatous inflammation *is mainly seen in TB and sarcoidosis. The pathologic feature is the formation of histiocytic granuloma in the lymph nodes.
5. *Acute lymphadenitis *is usually seen in the lymph nodes of the affected tissues involved in bacterial infection. Follicular hyperplasia and infiltration of polymorphonuclear (PMN) cells is the pathologic pattern. Suppurative adenitis smears show PMN and few lymphoid cells in a necrotic background.

Certain pathogens cause typical findings. Large transformed B immunoblasts, surrounded by some plasma cells with basophilic cytoplasm, are detected in Epstein-Barr virus infection. The features of the lymph node in Epstein-Barr virus involvement can be mistaken with Hodgkin's disease.^90^ The histological findings of cytomegalovirus lymphadenitis are similar to those of the Epstein-Barr virus, but large eosinophilic intranuclear inclusions are characteristically seen in cytomegalovirus. Mycobacterium TB produces a chronic specific granulomatous inflammation in which Langerhans' giant cells, caseating necrosis, and calcification can be seen.^91^ Satellite micro-abscesses, surrounded by granulomatous inflammation, are the hallmark of cat scratch disease.^92^Non-necrotizing epithelioid granuloma is a characteristic of sarcoidosis.^93^ The presence of Reed-Sternberg cells (a large cell with plentiful basophilic cytoplasm and prominent eosinophilic nucleoli) in a varied inflammatory cell infiltration background characteristically is seen in classical Hodgkin's disease.^88^

The histological patterns of Hodgkin's disease according to the World Health Organization (WHO) classification are:^94^ 1) nodular sclerosis; 2) lymphocyte-rich; 3) mixed cellularity; 4) lymphocyte-depleted; and 5) nodular lymphocyte-predominant. The principal histological subtypes vary by geographic location and economic level. In developed countries such as the US, nodular sclerosis Hodgkin's disease is the most common form of Hodgkin's disease (80%). It is most common in young adults, especially in women in poor economic areas.^94^ Mixed cellularity Hodgkin's disease is more common in children and older adults in developing countries.^4^^,^^11^

## Conclusion

Peripheral LAP is a common finding in routine clinical practice. When physicians are faced with it, the most serious task is to differentiate benign from malignant disorders. It is usually due to self-limited diseases, and most cases tend to subside without any sequel within a limited period, particularly in children. Some conditions require urgent attention and they include malignancy, TB, HIV infection, and immune-induced disorders such as systemic lupus erythematous, rheumatoid arthritis, and sarcoidosis. Special clues in the patient's history and physical findings can help to select suitable work-up for the patient.

In general, lymph nodes are considered abnormal if their diameter exceeds one cm. However, there is no uniform nodal size at which the greater diameter can raise suspicion for a neoplastic etiology. The cervical region is the most frequent site involved in peripheral LAP at any age. Generalized LAP usually is indicative of an underlying disease. Some important causes include the Epstein-Barr virus, HIV, lymphoma, and autoimmune disorders.

Ultrasound can assess the number, size, site, shape, margins, and pattern of vascularity and the internal structure of a lymph node. FNAC is more powerful in diagnosing metastatic cancers than lymphomas. Ultrasonography-guided FNAC offers more accurate information than does blinded FNAC. Needle biopsy can be used as the first step in the diagnostic approach to lymphomas, but excisional biopsy of enlarged lymph nodes is still the gold standard procedure.

Age more than 40 years, multiple sites of LAP, supraclavicular lymph nodes, nodal diameter greater than 2 cm, firm or hard texture, fixed nodes, lack of tenderness, and abnormal chest X-ray are factors that propel the physician into tissue sampling. If none of the predictive risks for malignancy is present, patients with peripheral LAP can be observed for 3 to 4 weeks before lymph node biopsy.
